# Multi-Sensor Data Fusion in A Real-Time Support System for On-Duty Firefighters

**DOI:** 10.3390/s19214746

**Published:** 2019-11-01

**Authors:** Van Thanh Pham, Quang Bon Le, Duc Anh Nguyen, Nhu Dinh Dang, Huu Tue Huynh, Duc Tan Tran

**Affiliations:** 1 Department of Electronics and Telecommunication, VNU University of Engineering and Technology, Hanoi 123000, Vietnam; phamvanthanh1209@gmail.com; 2 Department of Automation and Technical Equipment of Fire Fighting & Rescue, The University of Fire, Hanoi 100000, Vietnam; bonlequang.gov@gmail.com (Q.B.L.); anhpchp@gmail.com (D.A.N.); nhudinht34@gmail.com (N.D.D.); 3 School of Electrical Engineering, VNU International University; hhtue@hcmiu.edu.vn; 4 Faculty of Electrical and Electronic Engineering, Phenikaa University, Hanoi 12116, Vietnam; 5 Phenikaa Research and Technology Institute (PRATI), A&A Green Phoenix Group JSC, No.167 Hoang Ngan, Trung Hoa, Cau Giay, Hanoi 11313, Vietnam

**Keywords:** fall detection, loss of physical performance detection, barometer, firefighters, on-duty activities

## Abstract

While working on fire ground, firefighters risk their well-being in a state where any incident might cause not only injuries, but also fatality. They may be incapacitated by unpredicted falls due to floor cracks, holes, structure failure, gas explosion, exposure to toxic gases, or being stuck in narrow path, etc. Having acknowledged this need, in this study, we focus on developing an efficient portable system to detect firefighter’s falls, loss of physical performance, and alert high CO level by using a microcontroller carried by a firefighter with data fusion from a 3-DOF (degrees of freedom) accelerometer, 3-DOF gyroscope, 3-DOF magnetometer, barometer, and a MQ7 sensor using our proposed fall detection, loss of physical performance detection, and CO monitoring algorithms. By the combination of five sensors and highly efficient data fusion algorithms to observe the fall event, loss of physical performance, and detect high CO level, we can distinguish among falling, loss of physical performance, and the other on-duty activities (ODAs) such as standing, walking, running, jogging, crawling, climbing up/down stairs, and moving up/down in elevators. Signals from these sensors are sent to the microcontroller to detect fall, loss of physical performance, and alert high CO level. The proposed algorithms can achieve 100% of accuracy, specificity, and sensitivity in our experimental datasets and 97.96%, 100%, and 95.89% in public datasets in distinguishing between falls and ODAs activities, respectively. Furthermore, the proposed algorithm perfectly distinguishes between loss of physical performance and up/down movement in the elevator based on barometric data fusion. If a firefighter is unconscious following the fall or loss of physical performance, an alert message will be sent to their incident commander (IC) via the nRF224L01 module.

## 1. Introduction

**Figure 1:**
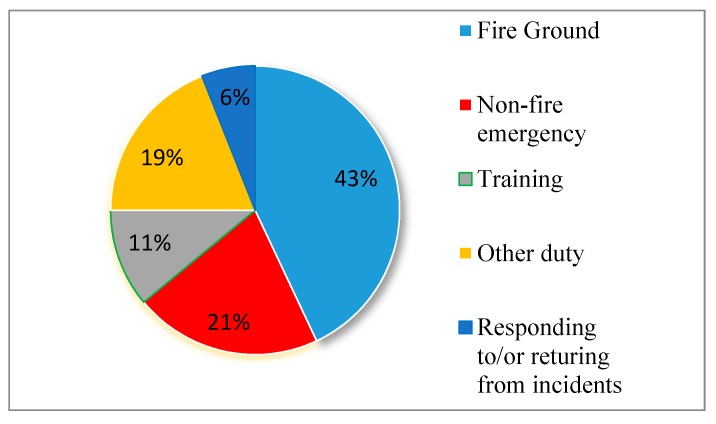
US firefighter injuries by type of duty during 2015 [1].

According to [1], 68,085 US cases of firefighter injuries were recorded in 2015 with 29,130 occurrences during fire ground operations and a total of 64 firefighters died on-duty in the same year [2]. The details of US firefighter injuries by type of duty during 2015 are illustrated in Figure 1. In Vietnam, thousands of fires on different scales occur every year; for example, 2357 and 2792 fires were reported in 2014 and 2015, respectively [3,4]. This is an alarming signal for firefighters in the US, Vietnam, and other countries over the world because they constantly work and are faced with perils yet possess inadequate suitable supporting systems to protect their lives. There are several supporting systems such as the fall detection system, PASS (Personal Alert Safety System). Nevertheless, most of the fall detection systems are only intended for the elderly and patients with slow movement, which are certainly inapplicable for firefighters’ activities in their working environment. The PASS system was developed by Homeland Security to signal for aid via an audible alarm if on-ground firefighter’s incapacitation is detected [5]. Furthermore, it can sense movement or loss of physical performance and activate a 95-decibel alarm if loss of physical performance exceeds a specific time period. However, in a real fire situation, there are a variety of noises like voices, cracking, sizzling of fire, the operation of fire protection systems, structure collapse, etc. Therefore, an audible alarm is not useful on a large scale. For instance, six and nine firefighters lost their lives in the fires in the Worcester cold storage and warehouse Co. at 266 Franklin street, Worcester, Massachusetts and The Charleston Sofa Super Store in Charleston, South Carolina respectively [6,7]. The proposed algorithms in this paper will be integrated with the indoor positioning system [8] to detect on-duty firefighters’ fall events and loss of physical performances to protect their health and lives.

Although a number of publications have proposed methods to detect the fall events [9] by the use of a camera [10,11,12,13,14], and accelerometer in combination with other support sensors [15,16,17,18,19]. Nevertheless, these publications mostly focus on detecting the fall events in the elderly or patients who walk slowly and execute low dynamic activities. Furthermore, using a camera for fall detection in fire conditions is not applicable because this is an invisible environment. Hence, this trend is insufficient in protecting firefighters who work on fire ground inside a building with physically demanding and complex activities.

The publication [17] proposes a waist-mounted system that combines three-axis accelerometer, three-axis gyroscope, three-axis magnetometer, and barometer with four features: Impact, Aftermath, Posture, and Altitude variation to detect a fall event. In addition, this research also mainly focuses on the elderly’s fall events. Using a barometer in fall detection is a novel contribution of this publication, but it still shows huge limitations. The accuracy of the algorithm may be decreased because of environmental noise, the wearing positions, volunteer’s height, and the kind of barometer, etc. Furthermore, the experimental data in testing results are mostly intended for the elderly. Hence, it will be a huge limitation when applying this method on other subjects, especially in firefighters.

The publication [20] (the Proetex project) proposed a system that integrates multi-sensors including external temperature sensor, heat flux sensor, accelerometers, motion sensor, CO sensor CO2 sensor, SPO2 sensor, heart, and breathing rate sensors. These sensors will record data and transmit data to the workstation through Wi-Fi module. The multi-sensor will monitor environment parameters effectively. Nevertheless, it will increase the cost of the system and complex computations. Furthermore, the fall detection algorithm in the Proetex project is still simple [21]. Hence, the performance of the system may decline in several situations such as crawling then falling as proposed in this paper.

The publication [22] proposed a wearable system to monitor the health of firefighters using the sensors to measure the respiration cycle and heartbeat. This method mainly focuses on monitoring physiological states of the users, yet it fails to keep track of firefighters’ activities or detect fall events in a fire ground. Hence, it cannot be applied to detect on-duty firefighters’ injuries. It is suitable to track the firefighter’s health state in normal conditions or during training.

To overcome the above deficiencies, this study proposes to develop a real-time, low-cost, and high-accuracy system using a 3-DOF accelerometer, 3-DOF gyroscope, 3-DOF magnetometer, barometer, and MQ7 sensor combined with development of the algorithms and the corresponding simulation process to support on-duty firefighters.

The paper has three novel contributions:

Firstly, the data fusion of three-axis accelerometer, three-axis gyroscope, and three-axis magnetometer has been proposed for fall detection in firefighters. Several publications have integrated these sensors for fall detection, but these methods mainly focus on detecting fall events for the elderly who do not perform any complex and strenuous activities and the thresholds within which to detect the fall events in firefighters are also different from those with the elderly. Furthermore, most of the datasets in previous publications were recorded from students, elders, or other volunteers other than firefighters. As a result, they are not really effective in detecting the fall events in the firefighters who perform complex and strenuous activities in fire environments.

Secondly, three proposed features comprising upper threshold, post-fall, and posture recognition are combined for fall detection. The combination of the theta (T) angle, pitch (P) angle, and roll (R) angle proves to be efficient. Furthermore, checking twice with an interval of 0.5 s in posture recognition feature enhances the accuracy of our proposed fall detection algorithm.

Thirdly, the proposed system has an integrated barometer to detect loss of physical performance because using 3-DOF accelerometer, 3-DOF gyroscope, and 3-DOF magnetometer to detect loss of physical performance is not strong enough. It will cause false warnings while the elevator is in use. Furthermore, long-time verification may cause death or permanent health damage in firefighters.

Finally, CO gas or the so-called “silent killer” gas is one of the most dangerous gases emitted from combustion. This gas can be seriously detrimental to firefighters’ health. Hence, the MQ7 sensor has been integrated into our proposed supporting system to optimize the use of self-contained breathing apparatuses (SCBA).

Our proposed system combines fall detection algorithm, loss of physical performance detection to confirm whether the user is suffering from fall, loss of physical performance or not. Moreover, it is also capable of using CO detection module to monitor the CO level on the fire ground. If the concentration is high, it then alerts firefighters to use SCBA. Otherwise, it will indicate the safety signal on the low concentration to save fresh air for more urgent situations.

## 2. Materials and Methods

In this section, the system architecture and the hardware components are described in detail.

### 2.1. System Architecture

**Figure 2:**
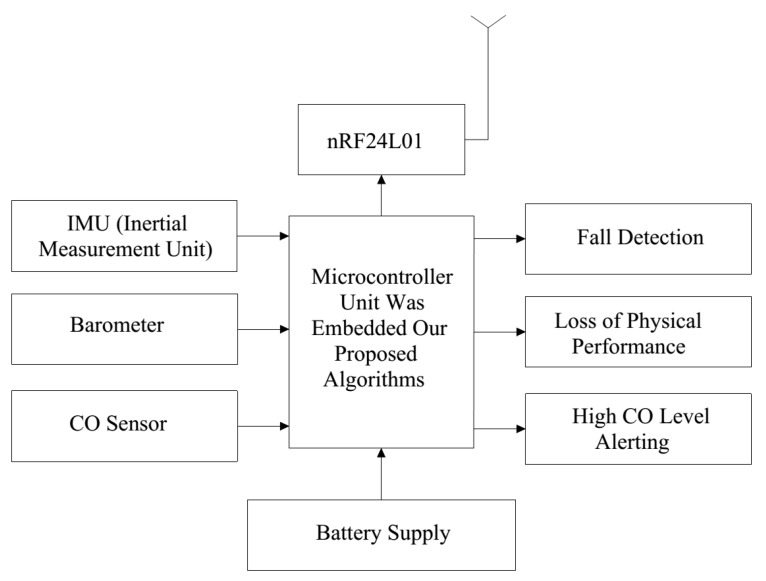
Block diagram of the proposed system.

Figure 2 shows the block diagram of the proposed system.

The 3-DOF accelerometer used in this paper collects data along Ax, Ay, and Az axes. The proposed system uses the I^2^C (inter-integrated circuit) interface in connection with a microcontroller unit with sampling rate of 100 Hz because the firefighter’s activities are quite quick.

The barometer is employed to collect pressure data; then the altitude will be calculated based on the measured pressure data. The altitude parameters will enable us to distinguish between loss of physical performance and other activities such as moving up/down the elevator. This is a novel contribution and an essential input parameter to enhance the performance and accuracy of our proposed support system.

The CO sensor is integrated in our proposed system to detect the CO level of environment in a structure fire. CO which is regularly called a “silent killer” is one of the most common and dangerous toxic gases in a structure fire while the amount of compressed air in a SCBA is of limited use, with about 30, 45, or 60 min [23]. CO sensor is used to guide the proper use of compressed air in a SCBA.

### 2.2. The 3-DOF Accelerometer

An accelerometer is the heart of our proposed system to detect the fall event of on-duty firefighters. The sensor is ADXL345, which can sense the acceleration along Ax, Ay, and Az axes. Output data is accessible through the I^2^C (inter-integrated circuit) digital interface. The accelerometer is positioned in the front trouser pocket on the earth frame so that the Az axis must be paralleled with the earth’s gravity to generate expected reading results of accelerometers approximately in [0, 0, 1] g (m/s^2^) with the rate of 100 samples per s. We applied a preprocessing phase before entering data into the attribute extraction module to formulate the mean, orientation, and standard deviation. The following is the equation used to calculate the measured acceleration:

(1) $$Acc_{\left(t\right)}=\sqrt{{\left({\mathrm{Ax}}_{\left(t\right)}\right)}^{2}+{\left({\mathrm{Ay}}_{\left(t\right)}\right)}^{2}+{\left({\mathrm{Az}}_{\left(t\right)}\right)}^{2}}$$

where, t = 0:1/Fs:end, Fs is the sampling frequency (Fs = 100 Hz), $Acc_{\left(t\right)}$ is the root mean square (RMS) of acceleration along Ax, Ay, and Az axes. The normalization of the measured 3-DOF acceleration data in g (g = 9.81 m/s^2^) is defined by the following equation:

(2) $$g_{\left(t\right)}=\frac{Acc_{\left(t\right)}}{9.81}$$

The preprocessing steps to eliminate abnormal parts in our measured data and calibration method have been proposed in our previous publication [18]. Thereby, we do not reintroduce them in this paper.

### 2.3. The Barometer

A barometer and accelerometer fusion enhance the accuracy and performance of our proposed algorithms. Furthermore, it is also used to eliminate “Alert message” in the case of loss of physical performance when a firefighter is moving the elevator up/down. This is extremely important and has not been proposed in the previous publications and market products.

The barometer is widely used to measure the pressure change of environment. Based on the measured environment pressure, the altitude can be determined by the equation below [24]:

(3) $$H_{k}=44330*\left(1-{\left(P_{k}/P_{0}\right)}^{\frac{1}{5.225}}\right)$$

where, k = 0:1/*Fs*:end; $H_{k}$ is the calculated altitude in meters, $P_{k}$ is the measured pressure, P_0_ is the pressure at the sea level (P_0_ = 1013.25 hPa).

When firefighters perform the tasks at the inside of a building, the barometer will measure barometric data. The change in pressures can be used to predict the state of firefighters. Nevertheless, the barometer is highly affected by systematic and environment noises in order to achieve highly accurate performance, we used the simple Kalman filter to eliminate the abnormal parts in the recorded barometric data. The details of the simple Kalman filter are as follows:

(4) $$K_{k}=\frac{P_{k-1}}{P_{k-1}+R}$$

where, $K_{k}$ is the Kalman gain; $P_{k-1}$ is the previous estimated uncertainty; R is the measured uncertainty. The update of the state is presented in the following:

(5) $$x_{k}=x_{k-1}+K_{k}\left(z_{k}-x_{k-1}\right)=x_{k-1}\left(1-K_{k}\right)+K_{k}z_{k}$$

where, $x_{k}$ is the current state, $x_{k-1}$ is the estimate of the signal on the previous state, $z_{k}$ is the measured value ($z_{k}\;\mathrm{is}\;H_{k}\mathrm{value}\;\mathrm{that}\;\mathrm{is}\;\mathrm{calculated}\;\mathrm{by}\;\mathrm{Equation}\;(3$). The update of estimation uncertainty is calculated by the following equation:

(6) $$P_{k}=\left(1-K_{k}\right)P_{k-1}+\left|x_{k-1}-x_{k}\right|*Q$$

where, $P_{k}$ is the current estimated uncertainty value.

**Figure 3:**
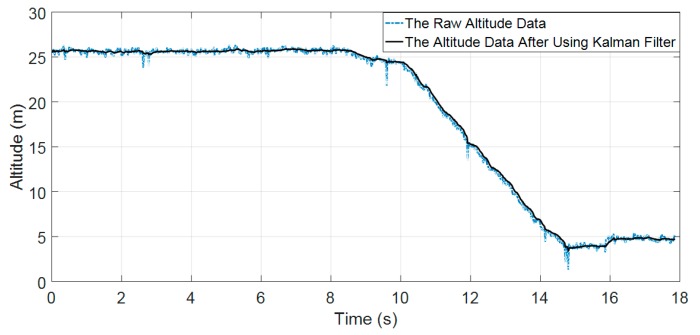
The comparison between the raw altitude signal and the altitude signal with the use of the simple Kalman filter.

Figure 3 shows the effectiveness of the simple Kalman filter in our experimental testing in eliminating the abnormal parts in barometric data. The application of the simple Kalman filter results in smoother recorded signal while characteristics of the signal remain.

### 2.4. The CO Detection

**Figure 4:**
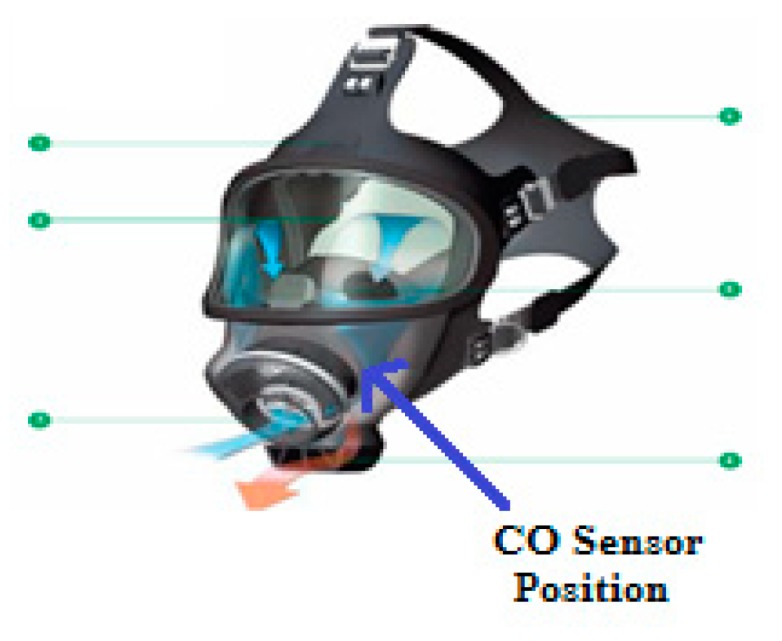
The position of CO sensor on the mask.

Numerous toxic gases are generated by the combustion process [25] such as: CO, CO_2_, NxO, NOx whose components depend on the type of burning materials. Nevertheless, CO is one of the most dangerous toxic gases [26] affecting firefighters as discussed above. We select a sensor named MQ7 to integrate in our proposed system for its popularity, low cost, high accuracy, low power consumption, and compatibility to our system. MQ7 sensor is mounted on the mask, as shown in Figure 4. Since the working time of the on-duty firefighters varies in each case depending on the scale of fire, along with various other factors, fresh air is considered one of the vital resources that have to be conserved. The detail parameters of MQ7 is presented in Table 1.

## 3. Method

**Figure 5:**
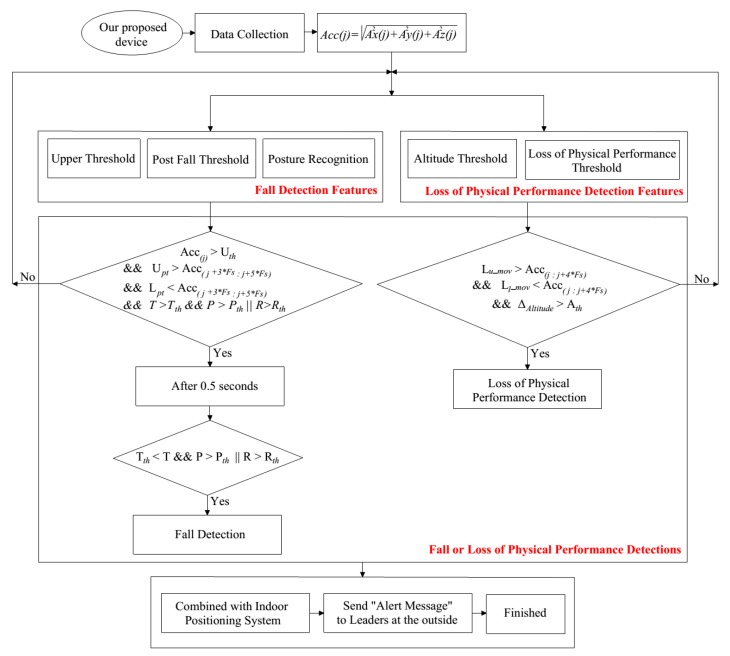
The flowchart for fall detection and loss of physical performance detection for firefighters.

Figure 5 is the flowchart for fall detection and detection for firefighters. It can be seen from the flowchart that the fall detection module consists of three features: Upper threshold, post-fall, and posture recognition. The loss of physical performance detection includes two main features: Altitude threshold and loss of physical performance threshold. The key condition for fall detection that is $Acc_{\left(j\right)}>U_{th}$ when RMS of acceleration exceeds the $U_{th}$ threshold value. In contrast, the loss of physical performance algorithm will work when $L_{u\_mov}>\left(Acc_{j}:Acc_{j+4*Fs}\right)>L_{l\_mov}$.

### 3.1. The Proposed Fall Detection Algorithm for Firefighter

**Figure 6:**
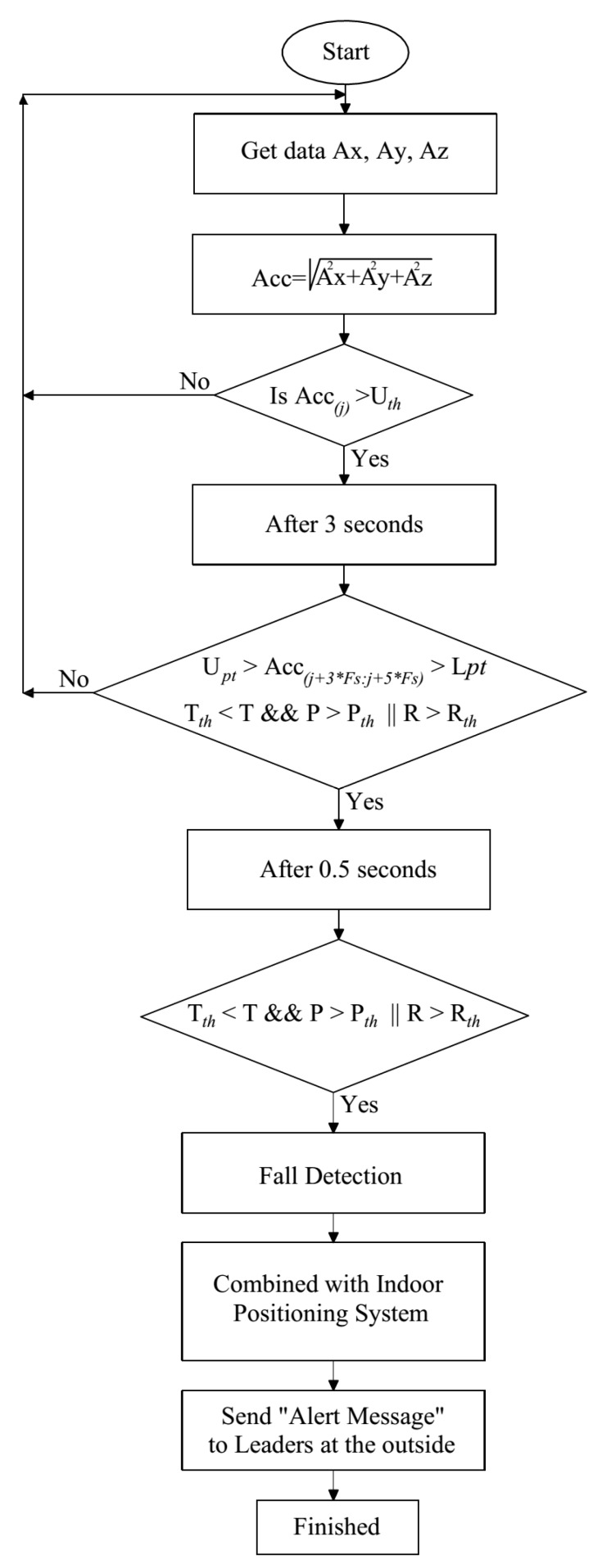
The proposed fall detection algorithm.

Figure 6 shows our proposed fall detection algorithm. The algorithm combines the use of three kinds of sensors: 3-DOF accelerometer, 3-DOF gyroscope, and 3-DOF magnetometer. The data fusion of these sensors has proved effective through our proposed algorithms. The difference among the fall detection algorithm in this paper, our previous fall detection algorithm, and other publications is the subjects. Most of the previous publications focus on detecting fall events in the elderly, children, or patients who obviously have less complex and lower dynamic activities than firefighters. In this paper, we focus on detecting the fall for firefighters who are working under fire conditions inside a building.

In our previous publication, we have proposed to use acceleration data and three modules (including Fall detection module, Posture recognition module, and Post-fall recognition module) to detect the fall events in the elderly with noticeable results. Nevertheless, this proposed method is used to detect falls of the elderly who have less complicated and physical demanding activities than those undertaken by firefighters. There are several scenarios that require strenuous activities such as running, walking, jogging while others just require moderate ones like scrawling, standing in elevator. Our previous publication and others do not cover any new activities and situations for firefighters such as crawling, crawling then falling. Therefore, the barometer has been integrated in our system to enhance the accuracy and performance of our proposed system. Four sensors will acquire data at the same time and with the same frequency. To detect the fall events in firefighters, we have proposed the three following features:

- **Upper Threshold.** At the fall state, after the volunteer’s loss of contact with the ground, their body will drop to “flight of fall” period. Due to the effect of gravity force, when the body initially contacts the ground or other objects, it will create a sudden change in acceleration data. Hence, using the upper threshold to detect the sudden increase in acceleration data has essential meaning. The upper threshold value also plays an important role in the accuracy and performance of the proposed system as well. In this research, we mainly focus on detecting the fall events for firefighters who are working in fire scenarios in buildings with high dynamics and low dynamic activities such as running, walking, crawling on the ground, or going up/down stairs.
  (7) $$Acc_{\left(j\right)}-U_{th}>0$$
  where, $Acc_{\left(j\right)}$ is the acceleration data at the sample of j; $U_{th}$ is the threshold to check the acceleration excess.
- **Post-Fall.** After the “flight of fall” period, the body will fluctuate in a short time before changing to the rest state. In the rest state, the RMS of accelerations in three axes is around 1 g. Postfall thresholds include upper and lower thresholds. Based on the experimental testing results, the rest state will be checked after the signal exceeds the upper threshold value by 3 s and the upper and lower thresholds of the post fall feature equal 1.25 and 0.75 g, respectively in this research. When RMS acceleration is greater and smaller than $L_{pt}$ and $U_{pt}$ thresholds within 2 s, respectively. The post-fall will confirm that a fall event has occurred.
  (8) $$\begin{array}{cc} {\mathrm{Pos}}_{fall}= & (U_{pt}>\left(Acc_{j+3*Fs}:Acc_{j+5*Fs}\right)\\ \&\&L_{pt}< & \left(Acc_{j+3*Fs}:Acc_{j+5*Fs}\right)) \end{array}$$
  where, $U_{pt}$ is the upper threshold to check post-fall condition, $L_{pt}$ is the lower threshold to check post-fall condition.
- **Posture Recognition.** After falling, the posture of the body will change. Hence, the roll, pitch, and yaw angles will change in comparison with the reference frame. In this research, the reference frame is the Earth frame. The changing of the posture means the changing in angles of roll, pitch, and yaw angles with the reference frame. The roll and pitch angles are used to estimate the posture of the firefighter after falling. Based on these, we proposed posture recognition threshold to distinguish between the on-duty activities and fall events.
  Furthermore, the theta (*T*) angle will change when the fall event occurs. Hence, the use of theta angle, pitch angle, and roll angle in the fall detection algorithm are of great importance in our proposed algorithm.

- **Condition 1:** The angle T between Az and gravity estimation:

The accelerometer is positioned in the front trouser pocket as Figure 10a. Thus, the T angle in the standing state is around 0°, and it changes when the device carrier is standing. The postures of the firefighter are detected using Equation (9) to determine the difference between T angle and the gravitational acceleration.

(9) $$T=\cos^{-1}\left(\frac{A_{z}\left(t\right)}{\sqrt{A_{x}^{2}\left(t\right)+A_{y}^{2}\left(t\right)+A_{z}^{2}\left(t\right)}}\right)\frac{180}{\pi }\;(\mathrm{degree})$$

The T angle is used to check the postures of the firefighter to confirm the fall event and remove fall positive events in our proposed system.

- **Condition 2:** The orientation estimation:

In this case, we will apply Madgwick orientation filter in eliminating noise for inertial measurement unit (IMU). The Euler angles represents the difference between the reference frame and the sensor frame defined by the following equations [28]:

(10) $$Y=Atan2\left(2q_{2}q_{3}-2q_{1}q_{4},\;2q_{1}^{2}+2q_{2}^{2}-1\right)$$

(11) $$P=-sin^{-1}\left(2q_{2}q_{4}+2q_{1}q_{3}\right)$$

(12) $$R=Atan2\left(2q_{3}q_{4}-2q_{1}q_{2},\;2q_{1}^{2}+2q_{4}^{2}-1\right)$$

where, Y, P, and R are the yaw, pitch, and roll of the Euler angles that rotate around the Az, Ay, and Ax axes of the reference frame, respectively.

Combination of Condition 1 and Condition 2:

In our proposed fall detection algorithm, the theta angle, pitch angle, and roll angle have been combined to enhance the sensitivity and accuracy of our proposed algorithm. Furthermore, the double check with an interval of 0.5 s also has been proposed in this paper to improve the accuracy of our proposed system. The detail of this combination is shown in Table 2.

Our support system is integrated with the indoor positioning system to track and locate firefighters for real-time alert in the case of injuries and send this message to the commander at the outside of the building. Then, based on the real-scenarios, the commander can decide the methods to rescue injured firefighters to safety.

As can be seen from Figure 6, when the Acc signal exceeds the upper threshold ($U_{th}$), after 3 s, the post-fall and posture recognition features are used to verify the fall event.

In the case of all these features being satisfied, the fall event will be confirmed, then the “Alert message” will be sent to the commander through the indoor positioning system. The fall event will be eliminated when one of these conditions is not met.

### 3.2. The Proposed Loss of Physical Performance Detection Algorithm for Firefighter

There are several kinds of fall events such as crawling then falling or getting stuck in narrow paths and spaces. The application of the previous algorithms to detect the accelerations that exceed the threshold are not applicable to these situations. The use of thresholds to detect the excess of acceleration may fail to detect firefighters’ injuries while the selection of a lower threshold value can increase the sensitivity of the proposed algorithm but the accuracy will decrease significantly and vice versa. Therefore, fall detection algorithms proposed in the previous publications are not very effective in these situations.

To solve these limitations, we have proposed the loss of physical performance algorithm. The loss of physical performance algorithm is an essential part of our proposed algorithm to detect accident events of firefighters. The data fusion between barometric and 3-DoF acceleration will help solve the existing limitations.

**Figure 7:**
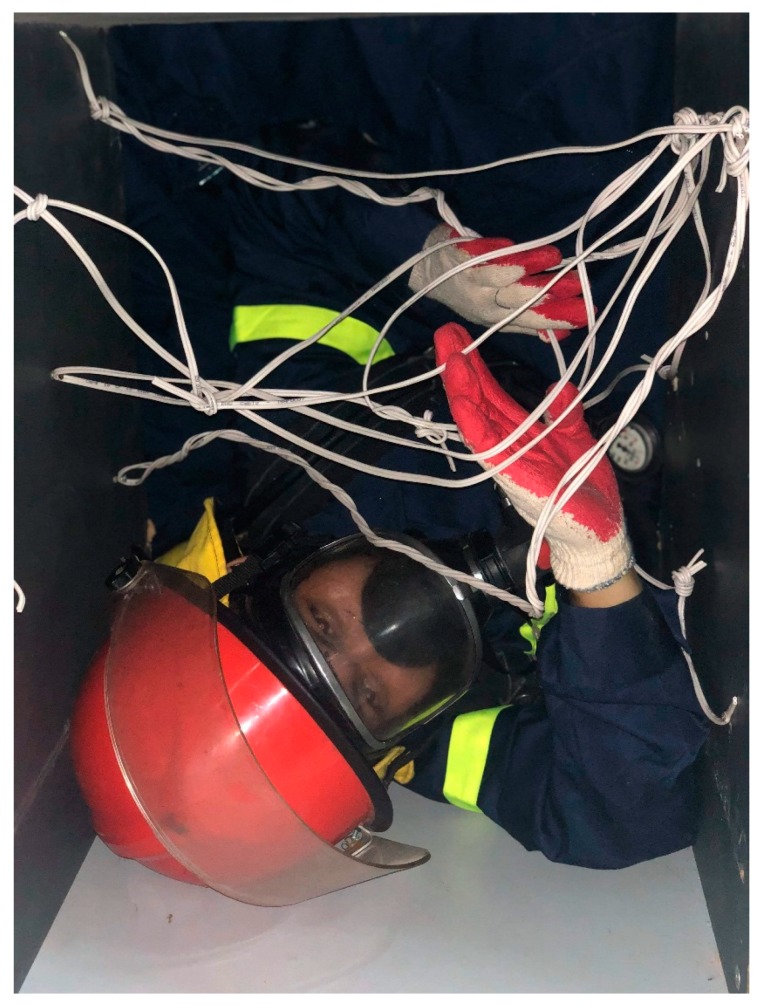
The narrow paths and spaces model.

**Figure 8:**
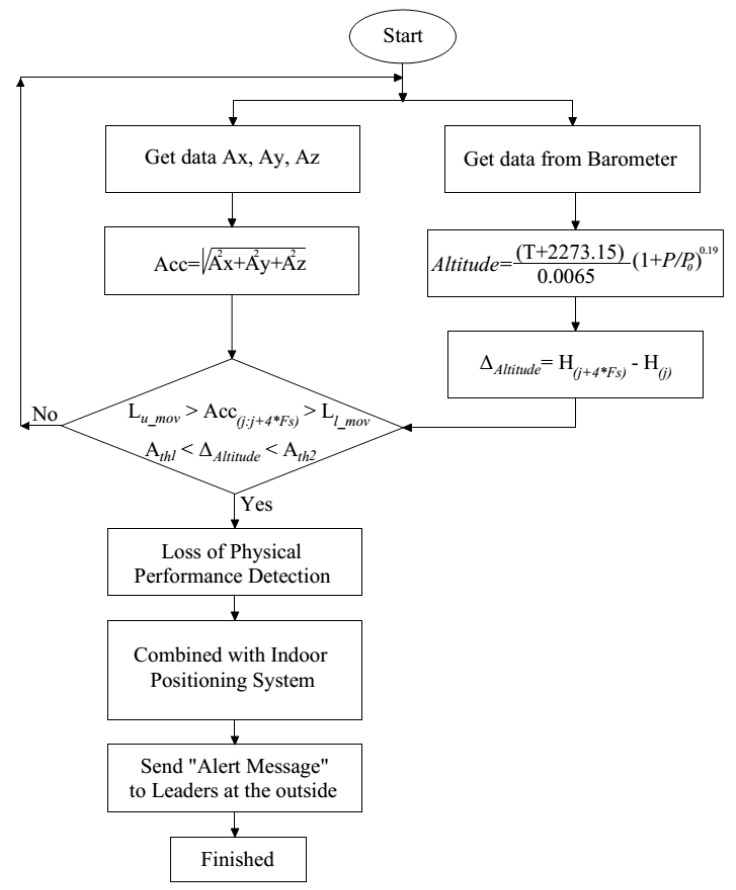
The loss of physical performance detection algorithm.

• **Loss of Physical Performance Threshold.** This threshold is applied as firefighters work in an invisible environment and need to pass through narrow paths and spaces, as shown in Figure 7:

The fall detection algorithm used only to detect the accident involving firefighters is not enough. Hence, the loss of physical performance feature is necessary in the case of being stuck or having an accident in these scenarios (see Figure 8). Based on Equation (18), when a firefighter is not moving or falling while crawling; the “Alert message” will be sent to the commander on the outside of the building to save his life. In this research, we propose that the loss of physical performance threshold value be equal to 4s.

(13) $$\begin{array}{c} L_{loss\;of\;physical\;performance}=\\ \left(L_{u_{mov}}>\left(Acc_{j}:Acc_{j+4*Fs}\right)\;\&\&\;L_{l\_mov}<\left(Acc_{j}:Acc_{j+4*Fs}\right)\right) \end{array}$$

• **Altitude Threshold.** The barometer is integrated in our proposed system and mounted in a front trouser pocket of volunteers. After using the simple Kalman filter to eliminate abnormal parts and the vibrations in data that is recorded from the barometer, the signal is quite clean and stable. When RMS accelerations satisfy Equation (13), the altitude information will be used to predict the state of volunteers.

(14) $$\Delta_{Altitude}=H_{\left(j+4*Fs\right)}-H_{\left(j\right)}$$

Based on the predicted result by fusion the data of both barometer and 3-DOF accelerometer, the loss of physical performance event will be confirmed when Equation (18) is satisfied.

(15) Predict(l)=

(16) $$Acc\langle L_{m}\;within\;T\;\&\Delta_{Altitude}\rangle A_{th}\;=>l=moving\;up$$

(17) $$Acc<L_{m}\;within\;T\;\&\Delta_{Altitude}<A_{th}\;=>l=moving\;down$$

(18) $$Acc<L_{m}\;within\;T\;\&A_{th1}<\Delta_{Altitude}<A_{th2}\;=>l=loss\_of\_physical\_performance$$

Furthermore, the results of Equation (16) and Equation (17) are used to estimate the floor in our proposed indoor positioning system.

### 3.3. CO Detection Algorithm for Firefighter

**Figure 9:**
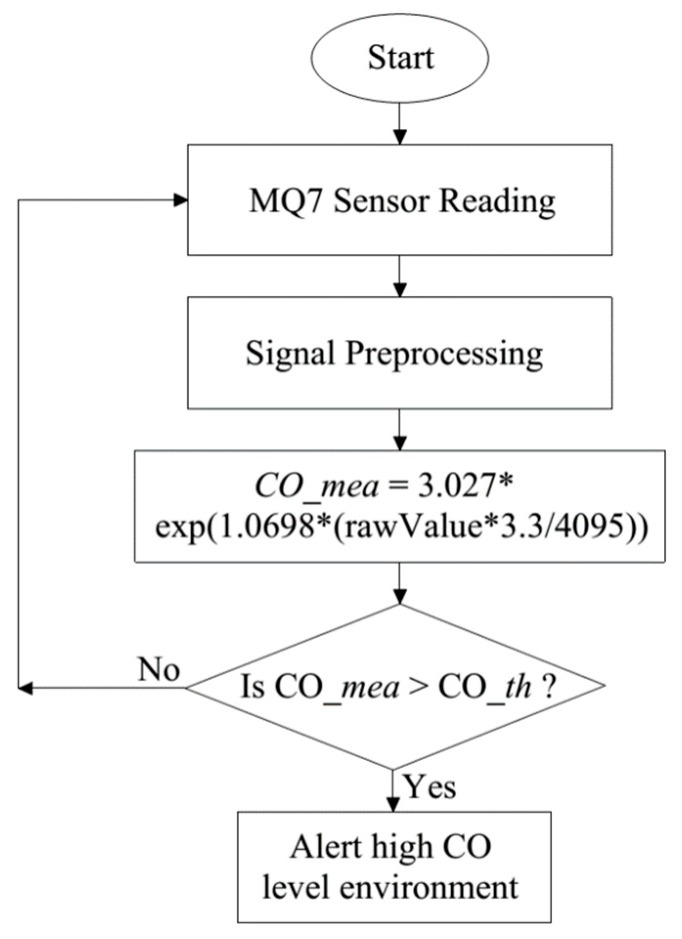
The high CO level alerting algorithm.

After being recorded, the raw data will be preprocessed to eliminate abnormal parts in the signals. The simple Kalman filter is also used to detect the CO level in our proposed CO detection algorithm, as shown in Figure 9.

As presented in Figure 9, the MQ7 sensor will be used to record CO data after calibration. Blood Carboxyhemoglobin (COHb) levels corresponding to adverse health effects of CO [26]. The CO data will be preprocessed then compared with the proposed threshold which is achieved through the statistical analysis and signs and symptoms of COHb level as shown in Table 3 that published in [26].

## 4. Result and Discussion

### 4.1. The Experimental Results

For the experimental setup, we tested the following:

The volunteers were randomly selected from many firefighters in The University of Fire—Vietnam. The volunteers executed the experiment to record data three times. The details of volunteer information are described in Table 4. They carried the devices with the CO sensor being mounted on the outside of the mask (see Figure 4).

On-duty firefighter activities are different from those executed by others like the elderly, patients, etc. In this paper, we focus on distinguishing among fall events, loss of physical performance, and other activities that include running, walking, jumping, jogging, crawling, and standing.

**Figure 10:**
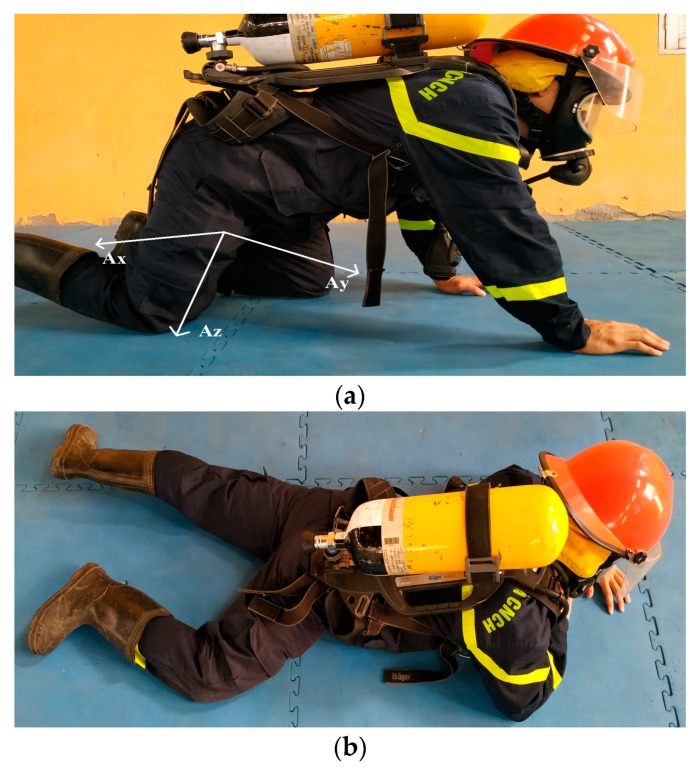
The volunteer is carrying the support device in his trouser pocket in the crawling state with the side view (**a**) and the down view (**b**).

Figure 10 illustrates the crawling activities of a firefighter with our proposed device in a trouser pocket.

Table 5 is the detail of the parameter values that were used in our proposed algorithms.

#### 4.1.1. Fall Detection Results 

**Figure 11:**
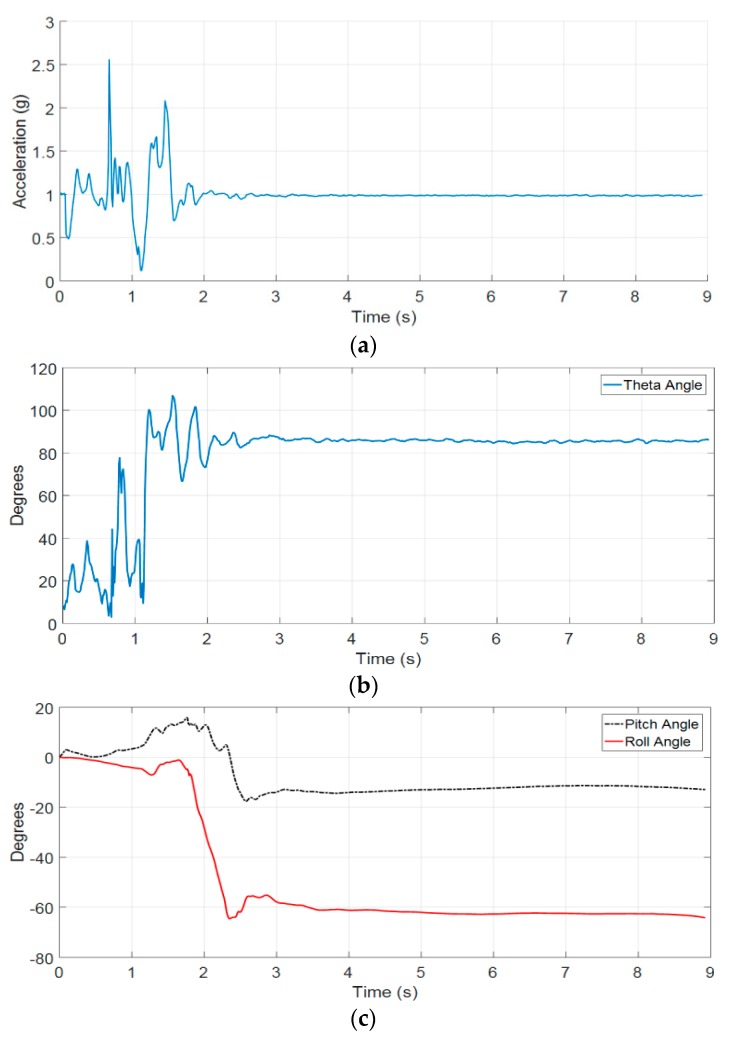
(**a**) The RMS of acceleration of a fall forward from standing, first impact on knees; (**b**) the theta angle; (**c**) the pitch and roll angles.

Figure 11 is the root mean square (RMS) of acceleration data along Ax, Ay, and Az axes. It can be seen that when the fall event occurred, the magnitude of signal suddenly changed until the body reached the ground. As the body contacts the ground, it will fluctuate slightly before changing to the rest state. Hence, our proposed fall detection algorithm uses the upper threshold to detect the sudden change in 3-DOF acceleration signal and post fall threshold to detect the rest state. Furthermore, the posture feature is integrated in our proposed fall detection algorithm to enhance the performance of our system.

After 3 s from the moment the upper threshold is detected, the post fall threshold condition will verify the fall event in the post fall state. The reason for selecting the delayed time of 3 s after the first condition is confirmed to ensure that the volunteer has become stable after having contacted the ground or an object.

#### 4.1.2. Loss of Physical Performance Detection

During the firefighting and rescuing process in a building/tower, the firefighter may need to use the elevator to move between floors. The application of PASS device may cause a false alert. Hence, our proposed support system for on-duty firefighters has considered loss of physical performance in firefighters while they are undertaking their tasks because they are subject to high risks of being injured or killed. The loss of physical performance algorithm will distinguish between the use of the elevator and accident by accelerometer data and barometer data fusion.

**Figure 12:**
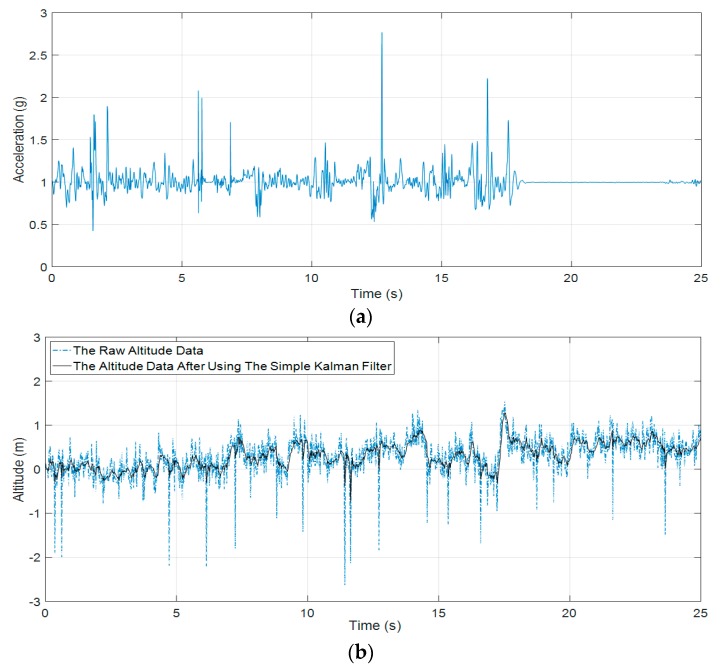
The loss of physical performance because of the accident (crawling then falling); (**a**) the RMS of accelerometer data; (**b**) the barometric data.

**Figure 13:**
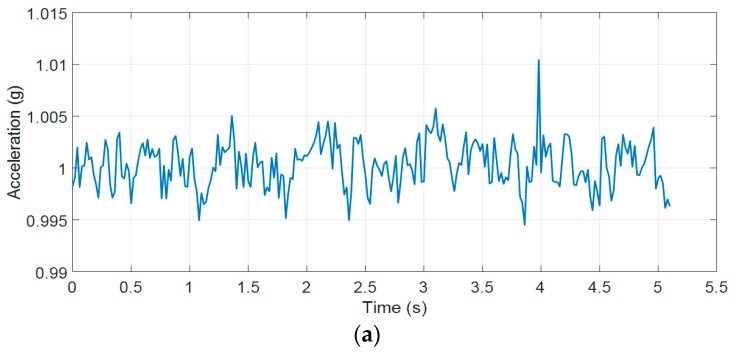

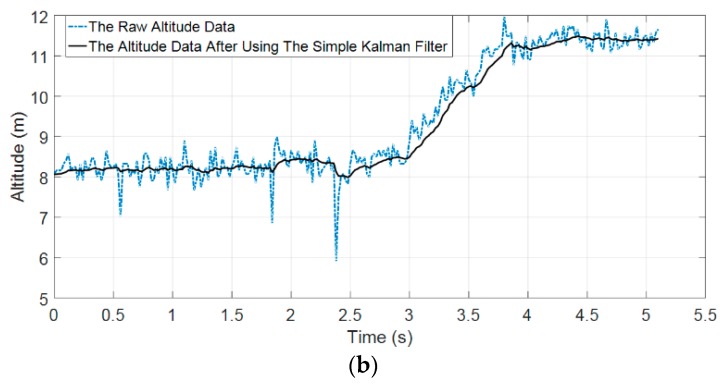
The loss of physical performance because of moving up in an elevator; (**a**) the RMS of accelerometer data; (**b**) the barometric data.

Figure 12 below illustrates the difference between the loss of physical performance because of being stuck or facing an accident and moving up in an elevator. As shown in Figure 12, both of the recorded datasets from the accelerometer and barometer are constant or show little change when a firefighter passes through a narrow path and they get stuck or have an accident. Similarly, Figure 13 shows the loss of physical performance because of moving up in an elevator.

In the case of moving in an elevator, accelerometer datasets have little change while barometer datasets increase or decrease depending on the change of environment pressure. Based on Equation (15), we can predict the state of the volunteer (firefighter) in this situation.

#### 4.1.3. High CO Level Alerting Algorithm

**Figure 14:**
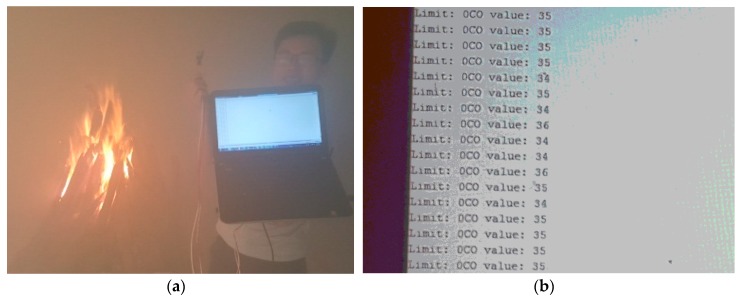
(**a**) Testing and measuring the CO level in the fire; (**b**) the measured CO values.

In Figure 14 experimental tests, the real-time CO measurement in smoke-filled room is shown in ppm (parts per millions). In the clean air (without a fire burning), this value only varied around 7 ppm, but it escalated rapidly when we moved the MQ7 sensor closer to the fire and the value varied from 33 to 45 ppm (see Figure 14b) when the sensor was located in the room.

Based on the empirical test at the University of Fire (Vietnam) and comparison to signs/symptoms listed in [26], we can confirm that when carbon monoxide concentration is around 35 ppm, headache and dizziness happen within 6 to 8 h of constant exposure. Moreover, the CO threshold for a hazardous work environment provided by the US OSHA CFR (U.S. Occupational Safety and Health Administration Code of Federal Regulations) in [27] is also 35 ppm. Hence, the authors propose using th5 = 33 ppm for alerting. If the CO level in a fire is lower than th5 threshold, firefighters can remove the breathing apparatus to save compressed air in a SCBA in case of a more serious situation ahead.

In addition to CO gas, other gases such as aldehyde, fine particles, H_2_S, HCN, CO_2_, NO_2_, N_2_O that are generated by a fire also affect firefighters’ health. For example, aldehydes are associated with numerous diseases such as cancer, respiratory allergies, cardiovascular, cerebral ischemia, neurodegenerative diseases, and stroke [29]. Fine particles are the causes of respiratory diseases, cardiovascular diseases, and cancer [30]. The proposed system in this paper is a partial result of our project to develop a real-time system to detect and track on-duty injured firefighters. Hence, the next version of our proposed system will integrate the sensors to detect aldehyde, fine particles, CO_2_, HCN.

### 4.2. Comparison

- Our previous publication mainly focuses on fall detection for the elderly. As a result, a lot of on-duty firefighter activities have not been considered in this research.
- Our current proposed method in a real-time support system for on-duty firefighters are a combined use of five sensors: A three-axis accelerometer, three-axis gyroscope, three-axis magnetometer, barometer, and MQ7 sensor. The efficient data fusion algorithms have been proposed for both fall detection and loss of physical performance detection with a noticeable performance in comparison with other public studies and marketed devices.
- Paola Pierleoni et al. [17] proposed an efficient fall detection algorithm by the combination of four kinds of sensors comprising a three-axis accelerometer, three-axis gyroscope, three-axis magnetometer, and a barometer. This method detected most of fall events of firefighters. Nevertheless, the case of crawling then falling and loss of physical performance have not been considered in this research because it mainly focuses on detecting fall event for the elderly while our proposed method has been developed for on-duty firefighters who carry out activities different from elderly’s.

In the comparison, we considered algorithms including:

- Algorithm 1: Our full algorithm with the features shown in Table 6.
- Algorithm 2: The reduced version of algorithm 1 (without checking theta, pitch, and roll angles at the second stage).
- Algorithm 3: The reduced version of algorithm 2 (without condition 1).
- Algorithm 4: The reduced version of algorithm 2 (without condition 2).
- Our previous fall detection algorithm.
- Paola Pierleoni et al. algorithm.

#### 4.2.1. The Comparison on Our Experimental Data

**Figure 15:**
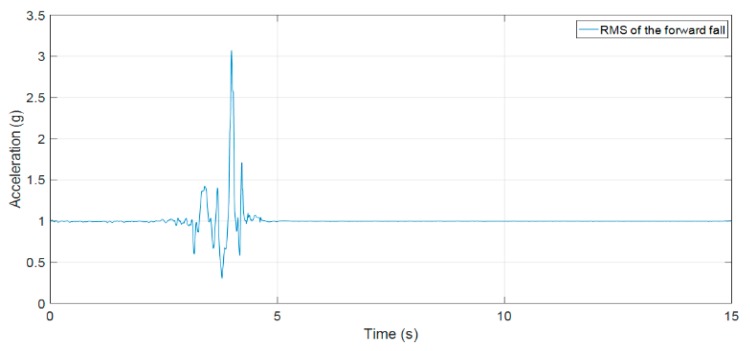
The RMS of acceleration of a fall forward from standing.

**Figure 16:**
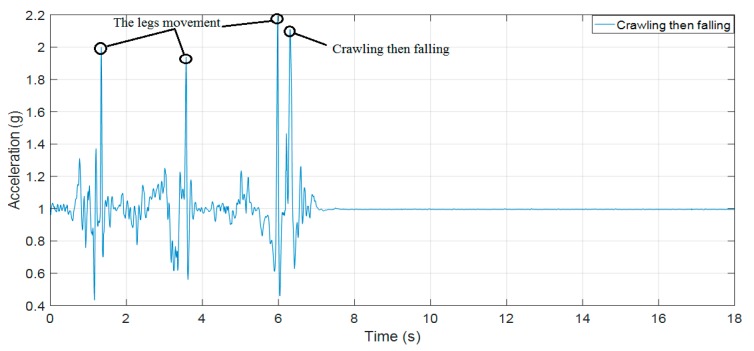
The RMS of acceleration of crawling then falling as the scenario of Figure 10a.

**Figure 17:**
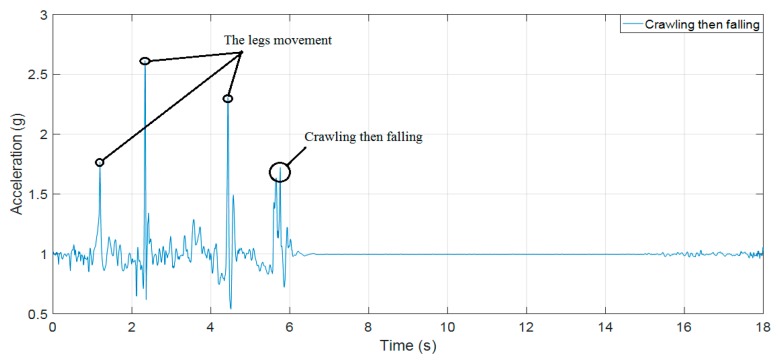
The RMS of acceleration of crawling then falling as the scenario of Figure 10b.

Figure 15, Figure 16 and Figure 17 clearly show results that our previous publication 18 and Paola Pierleoni et al. [17] can detect the fall events from standing, walking, jogging, or running with high accuracy because the sudden change in accelerations are strong enough to exceed the proposed threshold (the UFT threshold in [18] and Impact threshold in [17]). For the crawling characteristics, these publications still show some limitations because the fall signals of crawling as in Figure 16 and Figure 17 are lower than the leg movement signals. The direct application of these algorithms in fall detection for firefighters may fail to detect injuries. Consequently, on-duty firefighters may face deaths or injuries in building fire scenarios.

To evaluate the accuracy, specificity, and sensitivity of our currently proposed algorithms, our previous publication and Paola Pierleoni et al., we used the following equations (Equations (19)–(21)):

(19) $$Sen=\frac{TP}{TP+FN}$$

(20) $$Spec=\frac{TN}{TN+FP}$$

(21) $$Acc=\frac{TP+TN}{TP+TN+FP+FN}$$

where, True positive (TP) factor to determine if a fall has occurred and the device can detect it, False Positive (FP) factor to determine if a normal activity can be identified as a fall; True Negative (TN) factor to determine if a fall-like event is verified as a normal activity, and False Negative (FN) factor to determine if a fall occurs, but the device cannot detect it [18].

As shown in Table 7, our current proposed algorithms (fall detection and loss of physical performance detection) detect both fall events and loss of physical performance events. In the case of falling or being stuck when the volunteers are crawling and the accelerations are not exceeding the proposed threshold, the loss of physical performance detection algorithm is effective. Furthermore, the barometer has been integrated in our proposed system to support for IMU in distinguishing between true loss of physical performance (loss of physical performance because of accidents, injuries because of being stuck), and false loss of physical performance (loss of physical performance because of elevator use).

#### 4.2.2. The Comparison on Public Datasets

We continue compare on two public datasets [31,32] with the features shown in Table 8. On the public datasets [31], we do not consider data for females, and some positions of sensors such as on the head, right wrist, right thigh, and chest because most of the firefighters are male and these sensor’s positions are not suitable for firefighters in movement. Two subjects are very different in the fall and non-fall actions. Hence, only waist sensor data are used to compare our current proposed algorithms (fall detection and loss of physical performance detection), the reduced versions of our proposed algorithm, our previous fall detection algorithm and Paola Pierleoni et al. algorithm. Furthermore, some kinds of fall and non-fall events in males are not also considered in this comparison, including:

***Non-Fall Actions:***
  + 811 sit-chair from vertical, to sitting with a certain acceleration onto a chair (hard surface)
  + 812 sit-sofa from vertical, to sitting with a certain acceleration onto a sofa (soft surface)
  + 814 sit-bed from vertical, to sitting with a certain acceleration onto a bed (soft surface)
  + 815 lying-bed, from vertical lying on the bed
  + 816 rising-bed, from lying to sitting
***Fall Actions:***
  + 903 front-knees, from vertical falling down on the knees
  + 909 back-sitting, from vertical falling on the floor, ending in sitting
  + 917 rolling-out-bed, from lying, rolling out of bed and walking on the floor

Furthermore, the following recovery from fall events in this public dataset will be confirmed as non-fall events in our proposed algorithm to reduce unnecessary alert to the outside when firefighters can stand up after falling:

+ 905 front-quick-recovery, from vertical falling on the floor and quick recovery
+ 914 right-recovery, from vertical falling on the floor with subsequent recovery
+ 916 left-recovery, from vertical falling on the floor with subsequent recovery

**Figure 18:**
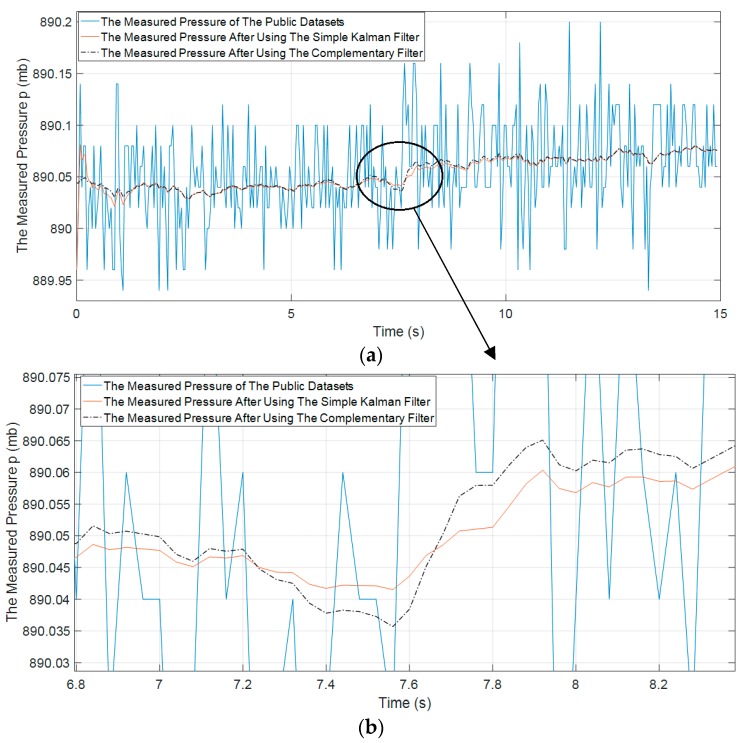
The measured pressure of the public dataset [31]; (**a**) the raw pressure data and estimated pressure data after using the simple Kalman filter and complementary filter, and (**b**) the zoom in raw pressure data and estimated pressure data after using the simple Kalman filter and complementary filter.

The proposed fall detection algorithm of Paola Pierleoni et al. [17] shows some limitations in testing with the public datasets [31] because the pressure datasets contain a lot of noises (see Figure 18a). Not many discrepancies exist in the pressure signal between before and after the fall event.

The performance of fall detection algorithms using barometric data is not stable enough because the barometer is strongly affected by environmental noises such as temperature and humidity. The pressure data of the public dataset [31] in a forward falling situation do not show clearly the fall event result between before and after the fall event has occurred (see Figure 18a,b).

Applying Equation (3) as proposed above we can calculate the altitude change in Figure 12 as follows:

(22) $$\begin{array}{cc} \Delta_{H}=\left|44330*\left(1-{\left(\frac{P_{T}}{P_{0}}\right)}^{\frac{1}{5.225}}\right)-44330*\left(1-{\left(\frac{P_{S}}{P_{0}}\right)}^{\frac{1}{5.225}}\right)\right|= & |44330*\left(1-{\left(\frac{890.0651}{P_{0}}\right)}^{\frac{1}{5.225}}\right)-\\ 44330*\left(1-{\left(\frac{890.0402}{P_{0}}\right)}^{\frac{1}{5.225}}\right)|\approx & 0.23\left(m\right) \end{array}$$

**Figure 19:**
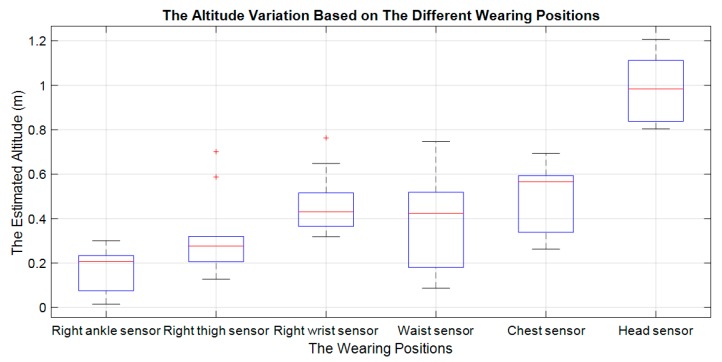
The altitude variations based on the different mounting positions of the fall events in public dataset 31.

Based on the public datasets, it can be seen that the maximum difference value is recorded between the current altitude and the estimated altitude after the Impact phase as proposed by Paola Pierleoni et al. [17]. The calculated altitude variation in the public datasets is much lower than the proposed altitude threshold value by Paola Pierleoni et al. (The altitude threshold for fall alert equals 0.52 m). This is an evidence to show the limitations of the use of the barometer in fall detection. It seems to be applicable to a specific system and subjects that the authors have proposed in their paper. Furthermore, the integrated barometer in the fall detection system also depends to large extent on the wearing positions and the end-user’s height, as shown in Figure 19.

Based on the testing performance in Table 9, it can be seen that our proposed algorithm (Algorithm 1) achieves better performance in terms of specificity and accuracy on public datasets. It can detect 762/762 non-fall actions and 724/755 fall actions equaling 100% and 97.96% in Table 9, respectively. Nevertheless, some kinds of fall such as the fall events in *920 syncope-wall, from standing falling down slowly slipping on a wall* in folders 107, 109, and 110 were declared as normal activities.

The fall detection results of our proposed algorithm on public datasets were added in the source: https://github.com/Pham-Van-Thanh/Fall-detection.

## 5. Conclusions

In this paper, we proposed the complete fall detection and loss of physical performance detection algorithms. These algorithms are fusing the data of 3-DoF accelerometer, 3-DoF gyroscope, 3-DoF magnetometer, and barometer. We have proposed the supporting system for a new subject which has not been focused on in previous publications that is the on-duty firefighter. This is the first time the loss of physical performance algorithm has been introduced to significantly enhance the accuracy of our supporting system. Furthermore, the paper also proposes using the suitable threshold value of CO in the fire to protect firefighter lives. The combination of five sensors and data fusion algorithms enable us to achieve noticeable results on a new subject as firefighter with high sensitivity, specificity of 100% and accuracy of 100% in our experimental datasets, and accuracy of 97.96% in public datasets. The research also shows the main kind of activities by firefighters in fire scenarios and proposes the suitable algorithms and threshold values through the use of five sensors and recording of data fusion of these sensors. For further research, we will integrate more toxic gas sensors to detect aldehyde, fine particles, CO_2_, and HCN to give a more precise decision on whether to use SCBA; and optimize our algorithms and thresholds in real-environments at the fire scene to save the life of firefighters.

## Figures and Tables

**Table 1:** The MQ7 sensor parameters [27].

Sensor	Symbol	Detecting Type of Gases	Range
CO	MQ7	CO	20–2000 ppm CO

The sensing level decreases from left to right order.

**Table 2:** Final decision based on the combination of theta angle, pitch angle, and roll angle.

Theta Angle (T)		Pitch Angle (P)		Roll Angle (R)		Final Decision
1st	2nd	1st	2nd	1st	2nd	
>$25^{{}^{\circ}}$	>$25^{{}^{\circ}}$	>$30^{{}^{\circ}}$	>$30^{{}^{\circ}}$	>$30^{{}^{\circ}}$	>$30^{{}^{\circ}}$	The fall occurred
One/some or all of them is/are smaller than their threshold.						The non-fall occurred

**Table 3:** Carbon monoxide concentrations, COHb levels, and symptoms 26.

Carbon Monoxide Concentration	COHb Level	Signs and Symptoms
35 ppm	<10%	Headache and dizziness within 6 to 8 h of constant exposure
100 ppm	>10%	Slight headache in 2 to 3 h
200 ppm	20%	Slight headache, fatigue within 2 to 3 h.
400 ppm	25%	Frontal headache within 1 to 2 h.
800 ppm	30%	Dizziness, nausea, and convulsions within 45 min; insensible within 2 h.
1600 ppm	40%	Headache, tachycardia, dizziness, and nausea within 20 min; death in less than 2 h.
3200 ppm	50%	Headache, dizziness, and nausea in 5 to 10 min; death within 30 min.
6400 ppm	60%	Headache and dizziness in 1 to 2 min; convulsions, respiratory arrest, and death in less than 20 min.
12,800 ppm	>70%	Death in less than 3 min.

**Table 4:** The volunteer characteristics.

Number of Volunteers	The Trial Times	Gender	Age	Height	Weight
5	3	Male	18–35	1.68–1.75	62–75 kg

**Table 5:** The parameter setting.

The Symbol	The Value	The Meaning
$U_{th}$	1.8 g	The threshold to check if the acceleration exceeds
$U_{pt}$	1.25 g	The upper threshold to check post-fall condition
$L_{pt}$	0.75 g	The lower threshold to check post-fall condition
T	25°	The theta threshold angle to check Posture Recognition condition
P	30°	The pitch threshold angle to check Posture Recognition condition
R	30°	The roll threshold angle to check Posture Recognition condition
$\Delta_{Altitude}$	0.5 m	The altitude threshold to confirm loss of physical performance condition
$L_{u\_mov}$	1.2 g	The upper threshold to check loss of physical performance condition
$L_{l\_mov}$	0.8 g	The lower threshold to check loss of physical performance condition
CO_th	35 ppm	The threshold to check high CO concentration environment

**Table 6:** The features of our experimental datasets.

	Our Experimental Datasets
Falls	Forward fall, Backward fall, Lateral left fall, Lateral right fall
OADs	Walking on the floor, Running on the floor, Crawling on the floor; Walking stairs up, Walking stairs down; Running stairs up, Running stairs down; Crawling stairs up, Crawling stairs down; Jumping, Taking the elevator up/down
Pos.	Pocket
Freq.	100 Hz
No. Vols	6

**Table 7:** The testing performance of our current proposed algorithms (fall detection and loss of physical performance detection), our previous fall detection algorithm, and Paola Pierleoni et al. algorithm on our experimental datasets.

The Algorithms Comparison	Sen	Spec	Acc
Algorithm 1	100%	100%	100%
Algorithm 2	100%	94.44%	95.83%
Algorithm 3	100%	90.74%	93.05%
Algorithm 4	100%	91.67%	93.75%
Our previous fall detection algorithm 18	88.9%	94.45%	91.67%
Paola Pierleoni et al. algorithm 17	66.7%	100%	83.33%

**Table 8:** The features of our the public datasets [31,32].

	The Public Datasets 31
Falls	901 front-lying, from vertical falling forward to the floor
	902 front-protecting-lying, from vertical falling forward to the floor with arm protection
	903 front-knees, from vertical falling down on the knees
	904 front-knees-lying, from vertical falling down on the knees and then lying on the floor
	905 front-quick-recovery, from vertical falling on the floor and quick recovery
	906 front-slow-recovery, from vertical falling on the floor and slow recovery
	907 front-right, from vertical falling down on the floor, ending in right lateral position
	908 front-left, from vertical falling down on the floor, ending in left lateral position
	909 back-sitting, from vertical falling on the floor, ending in sitting
	910 back-lying, from vertical falling on the floor, ending in lying
	911 back-right, from vertical falling on the floor, ending in lying in right lateral position
	912 back-left, from vertical falling on the floor, ending lying in left lateral position
	913 right-sideway, from vertical falling on the floor, ending in lying
	914 right-recovery, from vertical falling on the floor with subsequent recovery
	915 left-sideway, from vertical falling on the floor, ending lying
	916 left-recovery, from vertical falling on the floor with subsequent recovery
	917 rolling out of bed, from lying, rolling out of bed and going on the floor
	918 podium, from vertical standing on a podium going on the floor
	919 syncope, from standing falling on the floor following a vertical trajectory
	920 syncope-wall, from standing falling down slowly slipping on a wall
OADs	801 walking-fw, walking forward
	802 walking-bw, walking backward
	803 jogging, running
	804 squatting-down, squatting, then standing up
	805 bending, bending about 90 degrees
	806 bending-pick-up, bending to pick up an object on the floor
	807 limp, walking with a limp
	808 stumble, stumbling with recovery
	809 trip-over, bending while walking and then continuing walking
	810 coughing-sneezing, coughing or sneezing
	811 sit-chair from vertical, to sitting with a certain acceleration onto a chair (hard surface)
	812 sit-sofa from vertical, to sitting with a certain acceleration onto a sofa (soft surface)
	813 sit-air from vertical, to sitting in the air exploiting the muscles of legs
	814 sit-bed from vertical, to sitting with a certain acceleration onto a bed (soft surface)
	815 lying-bed, from vertical lying on the bed
	816 rising-bed, from lying to sitting
Pos.	340506 Head sensor
	340527 Chest sensor
	340535 Waist sensor
	340537 Right wrist sensor
	340539 Right thigh sensor
	340540 Right ankle sensor
Freq.	25 Hz
No. Vols	10

**Table 9:** The testing performance of our current proposed algorithms (fall detection and loss of physical performance detection), our previous fall detection algorithm, and Paola Pierleoni et al. algorithm on public datasets.

The Algorithms Comparison	Sen	Spec	Acc
Algorithm 1	95.89%	100%	97.96%
Algorithm 2	96.15%	98.42%	97.3%
Algorithm 3	97.75%	94.48%	96.11%
Algorithm 4	96.68%	93.43%	95.05%
Our previous fall detection algorithm 18	93.33%	91.67%	92.5%
Paola Pierleoni et al. algorithm 17	36.95%	97.76%	67.5%
